# *Yersinia* Phages and Food Safety

**DOI:** 10.3390/v11121105

**Published:** 2019-11-28

**Authors:** Carlos G. Leon-Velarde, Jin Woo Jun, Mikael Skurnik

**Affiliations:** 1 Agriculture and Food Laboratory, Laboratory Services Division, University of Guelph, Guelph, ON N1H 8J7, Canada; cleonvel@uoguelph.ca; 2 Department of Aquaculture, Korea National College of Agriculture and Fisheries, Jeonju 54874, Korea; advancewoo@snu.ac.kr; 3 Department of Bacteriology and Immunology, Medicum, Human Microbiome Research Program, Faculty of Medicine, University of Helsinki, 00014 HY Helsinki, Finland; 4 Division of Clinical Microbiology, HUSLAB, Helsinki University Hospital, 00029 HUS Helsinki, Finland

**Keywords:** *Yersinia enterocolitica*, bacteriophage, application of *Yersinia* phages, food safety, tail fiber protein, magnetic separation, biocontrol

## Abstract

One of the human- and animal-pathogenic species in genus *Yersinia* is *Yersinia enterocolitica*, a food-borne zoonotic pathogen that causes enteric infections, mesenteric lymphadenitis, and sometimes sequelae such as reactive arthritis and erythema nodosum. *Y. enterocolitica* is able to proliferate at 4 °C, making it dangerous if contaminated food products are stored under refrigeration. The most common source of *Y. enterocolitica* is raw pork meat. Microbiological detection of the bacteria from food products is hampered by its slow growth rate as other bacteria overgrow it. Bacteriophages can be exploited in several ways to increase food safety with regards to contamination by *Y. enterocolitica.* For example, *Yersinia* phages could be useful in keeping the contamination of food products under control, or, alternatively, the specificity of the phages could be exploited in developing rapid and sensitive diagnostic tools for the identification of the bacteria in food products. In this review, we will discuss the present state of the research on these topics.

## 1. Introduction

The most numerous organisms on Earth are the viruses of bacteria, bacteriophages (phages for short), that are present in 10-fold excess to bacteria, their host cells [1]. The total number of phages has been estimated to be around 10^31^ particles [2]. The constant arms range between phages and their prey, the bacteria, has generated high diversity among the phages in nature [3]. Phage research has, during the last ten years, seen a renaissance after several decades’ decline; the present interest focuses on genome and evolution research, on systems biology studies, and horizontal gene transfer. In addition, phages themselves are excellent tools in bacterial genetics, and phage-derived enzymes are indispensable tools in molecular biology. As the world is facing the threat of increasing antibiotic resistance, phage therapy, the 100-year-old remedy of bacterial infections practiced in the former Soviet Union and Russia, and also sporadically in Europe and the USA until the 1940′s, has been seen as a possible solution. In phage therapy, lytic phages have been preferred since, after replicating itself, the phage kills its host bacteria and does not disrupt the normal microbiota [4]. In addition to curing bacterial infections, phages can also be used prophylactically to control bacterial pathogens in food production, having the benefit that phages do not damage the populations of beneficial bacteria present [5]. 

## 2. Genus *Yersinia* and Diseases

*Yersinia pestis*, *Y. pseudotuberculosis,* and *Y. enterocolitica* constitute the human pathogenic species in the genus *Yersinia*; the remaining of the altogether 17 species of these facultative anaerobic Gram-negative coccobacilli are considered environmental non-pathogens. *Y. pestis* is highly virulent and the notorious causative agent of the bubonic plague. The other two, *Y. pseudotuberculosis*, and *Y. enterocolitica* are less virulent and cause a globally important foodborne zoonotic disease, known as yersiniosis. The symptoms of yersiniosis usually initiate with fever and abdominal pain. The bacteria have a tropism to lymph nodes and often cause painful mesenteric lymphadenitis easily confused with appendicitis. The infections are oftentimes manifested by diarrhea that can even be bloody [6]. As sequelae, mainly in human leukocyte antigen B27 positive individuals, yersiniosis may induce reactive arthritis or erythema nodosum. The pathogenicity of all *Yersinia* species depends on many essential virulence factors encoded by genes located both on the 70 kb virulence plasmid and on the chromosome. Among the over 50 recognized O-serotypes of *Y. enterocolitica*, only a few (predominantly O:3, O:5,27, O:8, and O:9) that carry the 70 kb virulence plasmid, are pathogenic [7], and are thereby, the major causative agents of yersiniosis [8]. While most *Y. enterocolitica* infections in humans are caused by consumption of raw or undercooked contaminated pork [8], rarely by contaminated raw milk or untreated water, the pathogen may also spread from person-to-person, or from infected animals or their feces [6].

*Y. enterocolitica* is considered an important zoonotic pathogen in developed countries, being the third most commonly reported in the European Union [9], and the cause of almost 117,000 illnesses and 35 deaths in the United States every year [6]. In Finland, with a population of ca. 5.5 million people, the reported number of yersiniosis cases is 500–600 annually (http://urn.fi/URN:ISBN:978-952-302-978-1). Furthermore, *Y. pseudotuberculosis* has recently caused several large outbreaks due to contaminated carrots and iceberg lettuce [10].

## 3. *Yersinia enterocolitica* Bacteriophages

Bacteriophages infecting *Y. enterocolitica* have been found to belong to all three-tailed families of the order *Caudovirales*, the *Myoviridae* (contractile tails), *Siphoviridae* (long, non-contractile tails), and *Podoviridae* (short, non-contractile tails). Initial studies from 1967 to 1987 were restricted to electron microscopy observations accompanied by some host range information of lytic phages used for typing *Y. enterocolitica* [11,12,13,14,15,16,17,18,19,20,21,22]. Further reports focused on the morphology and host range [23,24,25,26,27,28,29] interspersed with a more thorough characterization of several other *Y. enterocolitica* phages reporting on their genome sequence, proteome, morphology, host range, receptor specificity, and the identification of the receptor binding proteins (RBPs) involved in host recognition (Table 1).

### 3.1. Podoviruses

With the exception of phage φ80-18 [35], which utilizes the O-PS of *Yersinia enterocolitica* O:8 and O:7,8 and other strains that carry the O:8 moiety [35,45], all other Podoviruses that have been characterized, have been isolated using *Y. enterocolitica* O:3 strains as a host since it is the most predominant serotype involved in human yersiniosis. For example, in the European Union, the serotype O:3 accounts for over 89% of *Y. enterocolitica* infections followed by serotypes O:9 (7%), O:5,27 (2%), and O:8 (2%) [46].

Among these, the lytic phage φYeO3-12 isolated from raw sewage in Finland is perhaps the most thoroughly studied, displaying a marked specificity for *Y. enterocolitica* O:3 [30,47,48]. Likewise, the lytic phage vB_YenP_AP5 isolated from raw sewage in Canada with a high degree of sequence similarity to φYeO3-12 also exhibits a host range restricted to *Y. enterocolitica* O:3 [31,32]. Both of these phages utilize the O-PS of the LPS as the phage receptor [30,32,48], which is composed of a homopolymer of 6-deoxy-l-altropyranose [49,50,51]. These two phages also show activity against strains, which also contain 6-deoxy-l-altropyranose in their LPS such as *Y. enterocolitica* O:1 and O:2, as well as to *Y. mollareti* O:3 and *Y. frederiksenii* O:3. Specificity for serotype O:3 strains using the OP-S as a receptor has also been reported for the recently described lytic phage phiYe-F10 [33] and for several other phages described as the fPS Group 1 phage, and fPS Group 3 phages [28]. In contrast, fPS Group 2 phages utilize the OC of the LPS as the phage receptor [28], and phage φR8-01 utilizes the IC of the LPS [34]. Thus, their host range is narrower, and they do not infect *Y. enterocolitica* O:3 with a complete LPS chemotype [52]. Notably, for the majority of these phages, the phage tail associated receptor binding proteins (RBP) necessary for host cell recognition, adsorption, and initiation of infection have been identified. For example in phage φYeO3-12, the tail fiber protein derived from the product of gene 17 of 645 amino acid (aa) residues is considered the major host range determinant, since the replacement of the homologous gene in bacteriophage T3 with that of φYeO3-12 was sufficient to turn Enterobacteria phage T3 into a *Y. enterocolitica* infecting phage [30].

### 3.2. Siphoviruses

There are only two Siphoviruses reported to infect *Y. enterocolitica*. The most thoroughly characterized is the temperate phage PY54 that was isolated in Germany from farm manure. PY54 exhibits a host range with specificity for *Y. enterocolitica* of serotypes O:5, for the epidemiologically significant strains of serotype O:5,27 and some non-pathogenic strains of Biotype 1A [27,39,40,45]. Regrettably, the host cell receptor(s) of phage PY54 have yet to be identified. Moreover, the identification of the RBPs of phage PY54 is complicated by homology searches providing a sequence identity to numerous prophage genomes sequenced within bacterial genomes and having low similarity to other phage genomes. Still, in silico analysis of the PY54 genome reveals two probable RBP genes, *ORF22* and *ORF25*. Since the location of the probable RBP genes on the PY54 genome must be similar to the location of the related genes on the genomes of other lambdoid phages, alignment with the genome of *Enterobacteria* phage λ [NC_001416.1], suggests that *ORF22* and *ORF25* likely correspond to the phage λ host specificity protein J and side tail fiber (stf) protein, respectively [40,53,54]. In phage λ, the stf protein plays a role in primary attachment of the virion to the host, binding transiently until the tail tip, composed of the host specificity protein J, interacts irreversibly inducing structural changes in the tail, leading to viral DNA injection into the cell [55,56]. It is unclear, however, if the putative stf protein of PY54 is required since the corresponding protein in phage λ is not essential for adsorption to its receptor, the bacterial maltose pore protein LamB [57,58].

The other Siphovirus is *Yersinia* phage φR2-01, isolated in Finland using an O-PS negative LPS mutant derived from *Y. enterocolitica* O:8. We are in the process of characterizing its host range and RBP(s) and have identified BtuB as its receptor [37]. The phage φR2-01 genome presents a gene organization similar to that of *Enterobacteria* phage T5, which facilitates the identification of its putative RBPs by gene synteny and protein homology searches. In phage T5, the non-contractile tail contains a collar structure that also serves as a baseplate for three L-shaped fibers (pb1) and a cone-shaped tip structure (pb5) ending in a central tail fiber [59,60,61]. In the phage φR2-01, gene *123* corresponds to the terminal end of the L-shaped fiber (pb1), and the product of gene *144* is homologous to pb5 of phage T5, which binds to the *E. coli* Omp ferrichrome transporter FhuA [62,63].

### 3.3. Myoviruses

Among the *Y. enterocolitica* phages, the lytic phage PY100 isolated from farm manure in Germany displays the widest host range reported [64]. PY100 infects strains from the three human pathogenic species *Y. pestis*, *Y. pseudotuberculosis*, and *Y. enterocolitica*, as well as other *Yersinia* spp. including *Y. intermedia*, *Y. kristensenii*, *Y. frederiksenii*, and *Y. mollareti*. Among the *Y. enterocolitica* that are susceptible are strains belonging to serotypes O:3, O:5,27, O:8, O:9, several biotype 1A strains, and several untypeable strains [64]. PY100 has a small genome of linear dsDNA of 50,291 bp, in comparison to *Enterobacteria* phage T4, which has a genome that is 168 kb in size. More importantly, however, due to its exceptionally broad host range, PY100 is a candidate for use as a biocontrol agent [65].

In contrast, the extensively studied phage φR1-37 is the largest *Y. enterocolitica* phage that has been reported. Also, isolated from raw sewage in Finland, φR1-37 has a 262 kb genome in size with extended terminal redundancy and resembles morphologically *Pseudomonas aeruginosa* phage φKZ, which is considered one of the largest bacteriophages [41,66,67]. Phage φR1-37 has no overall DNA sequence identity with any other phage genome and codes for 366 putative gene products, most of which are unrelated to any known protein in databases, indicating that close relatives have not been characterized. Phage φR1-37 was isolated based on its ability to infect an O-PS negative derivative of *Y. enterocolitica* O:3 [41,68], and the φR1-37 host receptor has been determined to be the OC hexasaccharide [42,43]. Phage φR1-37 displays a broad specificity showing virulence for *Y. enterocolitica* O:1, O:3, O:5, O:5,27, O:6, O:6,31, O:9, O:21, O:25,26,44, O:41,43, O:41(27)43, and O:50 [41,43,69]. The phage receptor however, is also present in the OC of *Y. intermedia* O:52,54, and in the O-PS of *Y. similis*, which were also sensitive to φR1-37 [41,43,69]. Since the long-tail fiber genetic locus is highly conserved among the *Myoviridae* [70], the φR1-37 genome was inspected to locate genes encoding probable tail fiber proteins. The gene *g298* was identified as coding for a probable RBP homologous to the phage T4 long-tail fiber protein Gp37 involved in host recognition, and the adjacent gene *g297* presents homology to Gp38 from phage T4, a tail fiber assembly protein. Our unpublished results have confirmed that Gp298 and its chaperone Gp297 are involved in host recognition [71].

Next are *Yersinia* phage φR1-RT and vB_YenM_TG1, which are closely related Myoviruses with a T4-like gene arrangement isolated in Finland and Canada, respectively [44]. Based on phylogenetic analyses of their whole genome sequences and large terminase subunit protein sequences, a genus named *Tg1virus* within the family *Myoviridae* was established with TG1 and φR1-RT as member species by the International Committee on Taxonomy of Viruses (ICTV) [72]. These lytic bacteriophages exhibit a host range restricted to *Y. enterocolitica*, displaying virulence for strains of serotypes O:1, O:2, O:3, O:5, O:6, O:5,27, O:7,8, O:9, and some strains of serotype O:6,30 and O:6,31 at or below 25 °C. Phage adsorption analyses of LPS and *ompF* mutants demonstrated that the phage RBPs Gp12 (short-tail fiber) and Gp37 (long-tail fiber) use the LPS inner core heptosyl residues and the outer membrane protein OmpF as phage receptors, with the later acting as the primary host determinant. Analysis of RNA-sequencing data demonstrated that under different growth temperatures, there is an inverse correlation between the expression of *ompF* in the host and temperature. Consistently, quantitative proteomics data demonstrated that OmpF is maximally expressed at 4 °C, displays abundance at 22 °C, but is minimally expressed at 37 °C [44]. Collectively, these findings suggest the temperature-dependent infection of these phages is due to the strong repression of OmpF at 37 °C. Such an observation highlights the importance of thoroughly understanding phage-host cell interactions when considering the use of phages for biocontrol or diagnostic applications.

More recently, Jun et al. [36] reported the isolation of three additional Myoviruses (fHe-Yen9-01, fHe-Yen9-02, and fHe-Yen9-03) from sewage in Finland capable of infecting *Y. enterocolitica*. Among these, phage fHe-Yen9-01 was characterized as it had the broadest host range lysing 65 of 106 (61.3%) of *Yersinia* strains tested and formed plaques on 53/81 (65.4%) of the *Y. enterocolitica* strains tested, including epidemiologically significant strains of serotype O:3, O:5,27, and O:9. The phage genome has a T4-like gene organization and is closely related to the aforementioned *Yersinia* phages φR1-RT and vB_YenM_TG1 [36]. In addition, fHe-Yen9-01 was shown to be stable across a pH range of 5–9 and did not contain genes related to lysogeny or associated with undesirable virulence factors that might preclude their use as biocontrol agents [36].

## 4. Applications of *Yersinia* Bacteriophages for Food Safety

At present, studies on the application of *Y. enterocolitica* phages for food safety are focused on expanding the search for phages with the potential for use in diagnostics [31], or as biocontrol agents to reduce or eliminate this foodborne pathogen from domestic animals or from potentially contaminated foods and fomites [36,64,65].

### 4.1. Biocontrol of Y. enterocolitica

Despite the technological development in food processing to control foodborne pathogens, food safety has been continuously an issue in the food industry. As a major cause of foodborne outbreaks, microbial contamination has received much public interest. We are now encountering changing lifestyles that could make the food safety issue complicated: the abundance of RTE foods and the growing interest of the public toward exotic foods. Considerable progress in phage development for the food industry has been made in the last decade, ever since the US-FDA approved the first phage-based product for use in foods in 2006 [73].

In many cases, foodborne pathogens originating from raw materials can cause the contamination of food products. In addition, their contamination can be caused by the manufacture or processing of the product [4]. For the successful application of phages in food safety, various factors should be taken into consideration, which have been reviewed extensively [74,75,76,77,78,79]. Mainly, the phages should be strongly lytic, stable within the environment they are to be used in, and have a broad host range (alone or in a cocktail) encompassing epidemiologically significant strains. Moreover, the genomes of these phages should not contain any undesirable laterally transferable genes that are related to bacterial toxins, pathogenicity, antibiotic resistance, and or lysogeny. In addition, phages have some advantages regarding their commercialization. First, phages are natural [74], and this makes them very attractive since this is one of the top priorities in the food industry. It is well known that many consumers show a preference for natural or minimally processed products. The second advantage is that no known harmful effects have been reported to date with the use of phages [74]. Therefore, there are no reasons to restrict their application, and as such, these phages can be utilized for each stage along the ‘farm to fork’ continuum. Lastly, phages do not produce any adverse organoleptic changes; they do not affect the taste, texture, smell, or color of food [74].

In a recent study, we assessed the efficacy of *Yersinia* phages against *Y. enterocolitica* in food products and on kitchen utensils [36]. Artificial contamination models were generated to mimic situations that are considered to be sources of yersiniosis. Raw pork, ready-to-eat (RTE) pork, and milk were artificially contaminated with *Y. enterocolitica* to reach an approximate bacterial inoculation level of 10^3^ CFU/g or mL. Thereafter, the food samples were treated with phage fHe-Yen9-01 at a concentration of 10^8^ pfu/g or mL and maintained at appropriate conditions (raw pork, 4 °C for 72 h; RTE pork, 26 °C for 12 h; milk, 4 °C for 72 h). It was found that phage treatment prevented bacterial growth throughout the experiments, with counts decreasing by 1–3 logs on food samples. In addition, we designed kitchen utensils, such as wooden and plastic cutting boards and knives, and artificial hands, which were then artificially contaminated with *Y. enterocolitica*, and then treated with phage (10^8^ pfu/cm^2^ or mL). The bacterial counts decreased by 1–2 logs from the original levels of ca 10^4^ CFU/cm^2^ or mL. This is similar to results obtained by Orquera et al., 2012, who reported a 2-log_10_ unit decrease in *Y. enterocolitica* from inoculated raw meat at 4 °C after 48 h with the broad host range *Yersinia* phage PY100 [67]. Altogether, these studies reveal that phages can be applied for the control of *Y. enterocolitica* in foods and on kitchen utensils, and serve as a model for the prevention of other, more serious foodborne infections.

### 4.2. Phage RBPs for Use in Diagnostics

Although numerous methods using bacteriophages for the rapid detection of food-borne pathogens have been proposed [80,81,82,83,84,85,86,87,88,89,90,91], analogous approaches are yet to be considered for the detection of pathogenic *Y. enterocolitica* from foods. These methods rely on native or genetically engineered phages for infection and lysis of target cells to measure detection of ATP release, detection of other bacterial cytoplasmic markers, measurement of impedance, or the activity or detection of reporter genes. However, since several phages with specificity for *Y. enterocolitica* serotypes of concern are now well characterized, the development of similar methods is feasible. Another potential approach is perhaps through the use of phage-derived adhesion proteins rather than whole phages, as demonstrated by the use of cell-wall-binding domains (CBDs) of bacteriophage endolysins for the detection of the Gram-positive foodborne pathogens such as *Listeria monocytogenes*, *Bacillus cereus,* and *Clostridium perfringens* [92,93,94]. However, the use of CBDs from endolysins derived from *Y. enterocolitica* may be limited due to the structural differences between the Gram-negative and Gram-positive cell walls, where in the latter, the peptidoglycan layer is readily accessible from outside the cell. Lastly, the RBPs of bacteriophages have also been proposed as ideal ligands for use in diagnostics since they are responsible for initial and specific interaction with host cell receptors.

**Figure 1:**
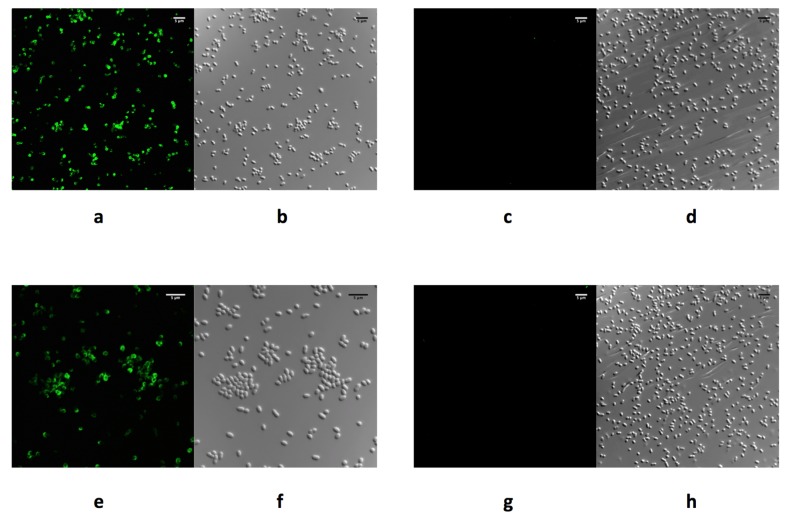
Confocal immunofluorescence microscopy images of *Y. enterocolitica* cells after incubation with Receptor Binding Proteins (RBPs) derived from phages vB_YenP_AP5 and φ80-18. Bacterial cells were exposed to N-terminal His_6_-tagged RBPs followed by immunolabelling with anti-His_6_ mouse monoclonal antibody and goat anti-mouse DyLight 488 conjugated secondary antibody. RBP Gp17 derived from phage vB_YenP_AP5 decorates the cell surface of a *Y. enterocolitica* strain YeO3 of serotype O:3 (**a**), while deletion of the phage receptor (O-PS) in the *Y. enterocolitica* strain YeO3-R2 abolishes cell decoration by RBP Gp17 (**c**). Similarly, in (**e**), RBP Gp47 derived from phage φ80-18 decorates the cell surface of the *Y. enterocolitica* strain 8081 of serotype O:8, whereas, the deletion of the phage receptor (O-PS) in the *Y. enterocolitica* strain 8081-R2 abolishes cell decoration by RBP Gp47 (**g**). Differential interference contrast microscopy of images of a, c, e, and g, are shown in b, d, f, and h, respectively. Scale bars represent the size in µm [31].

Recently, we successfully produced soluble purified recombinant forms of correctly folded phage RBPs Gp17, Gp47, and Gp37, derived from *Y. enterocolitica* phages vB_YenP_AP5, φ80-18, and vB_YenM_TG1, respectively, for use in diagnostics [31,44]. Analysis via confocal laser immunofluorescent microscopy confirmed the specific binding of purified recombinant forms of the RBPs Gp17, Gp47, and Gp37 to host cell surfaces (Figure 1), demonstrating a similar binding spectrum to the host range of the phages they were derived from based on the analysis of 165 *Yersinia* sp. strains [31,44]. In addition, to explore their potential use in biosensor applications, surface plasmon resonance (SPR) analyses of the interaction between these phage RBPs and *Y. enterocolitica* cells was possible following immobilization of the RBPs via an amine coupling procedure on a gold sensor chip surface. Analysis of the sensogram data indicated that the equilibrium association constant (K_A_) values were in the nanomolar range, which reflects an overall high affinity of the RBPs for host cells. Such binding affinity at the nanomolar level is in agreement with previous findings of Marti et al. (2013) [95], who investigated the interaction between the RBP of *Salmonella* phage S16 and wild-type *Salmonella* cells. Experiments showed that SPR signals were obtained after exposure of RBP Gp17 saturated gold sensor to different concentrations (10^6^, 10^5^, 10^4^, 10^3^, 10^2^ cfu/mL) of *Y. enterocolitica* O:3. The lowest signal for bacterial capture was achieved at a cell concentration of 10^3^ cfu/mL, compared to control samples that were not recognized. Similarly, a genetically engineered tail spike protein derived from *Salmonella* phage P22, anchored to a gold surface via an N-terminal cysteine tag, was able to detect *Salmonella* cells through SPR at a concentration of 10^3^ cfu/mL [96]. Likewise, an RBP from *Campylobacter* phage NCTC 12673 fused to glutathione S transferase (GST), and attached to an SPR surface using self-assembled glutathione monolayers, achieved a similarly low detection limit of 10^2^ cfu/mL [97]. These observations suggest the produced RBPs retain their biological function and represent highly specific reagents with an affinity for the predominant *Y. enterocolitica* serotypes implicated in yersinosis, and can be applied for use in diagnostics.

### 4.3. Agglutination Assays

An immediate application of RBPs is as an alternative to antibodies for the rapid screening of suspect colonies from culture isolation plates. RBPs are multimeric structures like antibodies; therefore, they are expected also to cross-link and agglutinate target bacterial cells [98,99]. To explore the agglutinating capability of Gp17, slide agglutination reactions were performed using individual *Y. enterocolitica* O:3 colonies obtained from growth on different agars used for routine isolation of this organism from contaminated foods, including MacConkey agar, Cefsulodin-irgasan-novobiocin (CIN) agar, *Salmonella*-*Shigella* agar supplemented with Sodium Desoxycholate and Calcium Chloride (SSDC), and CHROMagar *Yersinia* agar (CAY). Agglutination was observed within 5 min and was not affected by growth on these selective agars, whereas no agglutination reactions were observed with YeO3-R2 (an O-PS-deficient LPS mutant which lacks the phage receptor). The minimum agglutination concentration of RBP Gp17 resulting in cell agglutination was estimated at approximately 130 ng/mL and showed equivalent agglutination to that of *Y. enterocolitica* O:3 antisera [31].

### 4.4. Use of RBPs for the Selective Isolation of Y. enterocolitica from Foods

Present culture methods are considered inadequate and insufficiently sensitive to detect low levels of pathogenic *Y. enterocolitica* in foods, water, and environmental samples because they suffer from lengthy incubation times, cultural bias, and lack specificity [8,100,101]. As a result, numerous culture-independent methods have been devised for the rapid detection of *Y. enterocolitica* including: ELISA [102], lateral flow immunoassays [103], immunoblotting [104], DNA colony hybridization [105], PCR [106,107], nested PCR [108], real-time PCR [109], DNA microarrays [110], and loop-mediated isothermal amplification (LAMP) [111]. Among these, molecular-based methods have served to investigate the occurrence of pathogenic *Y. enterocolitica* in foods. Studies have shown the prevalence of naturally contaminated foods is significantly higher when estimated by PCR than by culture methods from epidemiologically relevant foods, highlighting the low sensitivity of culture methods [100,112,113]. Difficulties in isolation are largely due to low numbers of the organism present against a large number of background microbiota, and slower growth than other competing Gram-negative bacteria [8,114,115,116,117,118]. Specifically, different strains vary in tolerance to selective agents and incubation conditions during isolation [105,113,119,120]. Numerous approaches have been established utilizing combinations of cold pre-enrichment, potassium hydroxide (KOH) treatment, selective enrichments, and the use of varied selective and differential agars for isolation [8,100]. Taking into account all these challenges, alternative approaches for the cultural isolation of this organism from foods are required.

A promising approach is through selective capture via the use of immunomagnetic separation (IMS) [121]. Although IMS has been investigated previously as an aid for the isolation of pathogenic *Y. enterocolitica* from foods [122,123,124,125,126,127], the approach did not find acceptance for routine isolation. It is perhaps because immunological-based methods require antibodies produced by laboratory animals, which suffer from lot to lot heterogeneity and instability against environmental factors [128]. Furthermore, antisera to only a limited number of *Y. enterocolitica* serotypes are commercially available. Although the production of monoclonal antibodies (mAbs) with potentially higher specificity against *Y. enterocolitica* has also been explored, the similar bacterial surface structures within the genus make the selection of mAbs with high specificity against a particular serotype rather difficult and costly [104,129,130]. We sought then to investigate the use of magnetic microparticles functionalized with the phage RBPs Gp17, Gp47, or Gp37 as an alternative to antibodies for the isolation of *Y. enterocolitica* from foods. The aim was to utilize a phage RBP-based magnetic separation (RBP-MS) approach for the specific and simultaneous capture of the epidemiologically significant *Y. enterocolitica* serotypes O:3, O:5,27, O:8, and or O:9.

Selective capture of *Y. enterocolitica* of serotype O:3, the most significant serotype causing yersiniosis, was achieved from cell suspensions by use of magnetic microparticles coated with the RBP Gp17 derived from phage vB_YenP_AP5. However, the simultaneous capture of *Y. enterocolitica* O:3, O:5,27, O:8 or O:9, the predominant serotypes involved in yersiniosis, was attained utilizing a mixture of magnetic microparticles coated with RBPs Gp47 and Gp37 derived from phages φ80-18 and phage vB_YenM_TG1, respectively [31]. When applied to cell suspensions of 160 *Yersinia* strains and 20 other non-*Yersinia* spp. in combination with CIN agar, the strains isolated reflected the combined binding spectrum of the RBPs. Overall, the specificity obtained for the isolation of *Y. enterocolitica* O:3, O:5,27, O:8, and O:9 in combination with CIN agar was estimated at 85%. In contrast, the application of a mixture of RBP Gp47 and Gp37 coated microparticles in combination with CAY agar, a chromogenic medium selective for virulent *Y. enterocolitica*, increased the specificity to 95.7%. Pathogenic strains of serotype O:3, O:5,27, O:8 and O:9 were captured and grew as typical mauve colored colonies on this agar, whereas non-pathogenic strains grew as metallic blue or white translucent colonies including avirulent serotype O:7,8 strains that are rather common among biotype 1A strains [131] and O:6,30, O:6,31, which are isolated most often [132]. Only the rarely encountered pathogenic O:1 and O:2 strains, O:5, and O:6 strains, showed similar morphological characteristics to that of pathogenic O:3 strains on CAY agar [31]. More importantly, this approach prevented the appearance of confounding colonies that were observed with the use of CAY alone from the growth of some *Y. aleksiciae*, *Y. bercovieri*, *Y. kristensenii*, *Y. mollareti*, and *Y. rohdei* strains, as well as *Aeromonas hydrophila* and *Stenotrophomonas maltophilia* [31]. This is of significance since *Y. frederiksenii* and *Y. bercovieri* are considered the most commonly encountered species obtained during the routine culture isolation of *Y. enterocolitica* [133], and pose a challenge to the routine application of chromogenic agars since they appear indistinguishable from pathogenic *Y. enterocolitica* [134,135,136].

**Figure 2:**
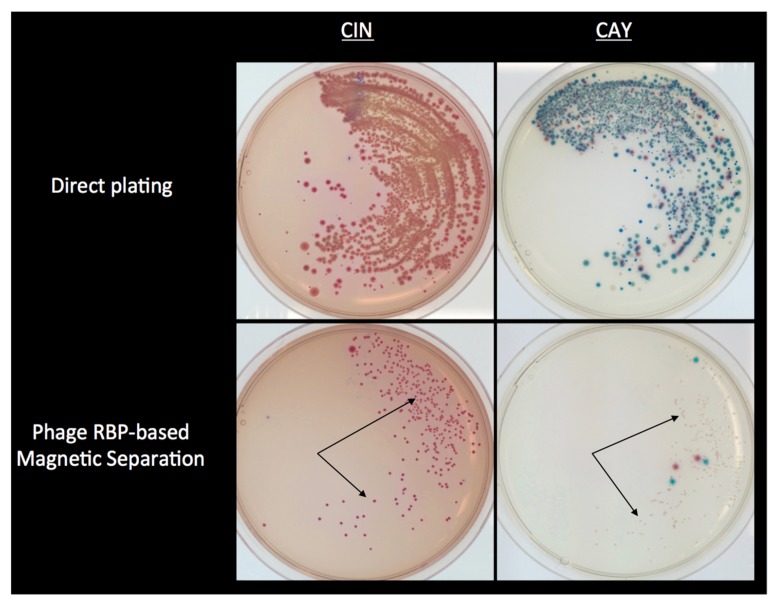
Effect of phage RBP-based magnetic separation in combination with CIN and CAY agar. A mixed cell suspension containing *Y. enterocolitica* O:3 cells (10^4^ CFU/mL) at a 1:9 ratio to other competing strains (each at 10^4^ CFU/mL) was reacted with RBP Gp17-coated magnetic microparticles and plated on Cefsulodin-irgasan-novobiocin (CIN) and CHROMagar *Yersinia* (CAY) agars. After incubation for 24 h at 30 °C and based on the colony morphology of these agars (arrows indicate *Y. enterocolitica* colonies), few competing organisms could be found compared to the growth observed with the use of direct plating alone (top) [31].

Lastly, when RBP-MS was applied to artificially inoculated ground pork samples homogenized in PBS, it established a 2-log_10_ cfu/g improvement in sensitivity compared to direct plating on CIN or CAY agars alone [31]. In addition, RBP-MS, in combination with CAY agar, was able to detect a higher number of *Y. enterocolitica* in ground pork, mixed salad, and milk samples inoculated at low levels (0.1 cfu/g, 1 cfu/g, and 10 cfu/g) after 8 h and 24 h enrichment at 25 °C in non-selective TSB. Particularly, a marked decrease in background microbiota on the isolation plates was observed as compared to those from samples that did not undergo RBP-MS (Figure 2).

In summary, RBP-based magnetic separation permits the separation of the target bacteria from competing microorganisms present in a food matrix. Unlike antibodies, however, RBPs due to their structural characteristics also offer distinct advantages such as stability to extreme pH values and temperature [89]. The use of phage RBPs as capture ligands represent an alternative approach for bacterial concentration that could also be used to improve the analytical sensitivity of rapid methods of analysis such as PCR or ELISA, as well as for the use in emerging biosensor based technologies. In addition, unlike intact phages, the use of RBPs provides a novel family of diagnostics that can be engineered and produced in vitro using recombinant DNA technologies, without the involvement of the phage-pathogen propagation system and the risk of cell lysis or gene transduction [89,90,91,92,93,94,95,96,97].

## 5. Perspectives

### 5.1. Diversity of Yersinia Enterocolitica Phages

The overview of the phages presented herein highlights the limited number of *Y. enterocolitica* phages for use in food safety applications. For example, among the phages examined, there is only one serotype O:8 specific phage (φ80-18) reported and no serotype O:9 specific phages. Comparatively, Salem et al. [29] reported the isolation of a high frequency of O:3 specific phages and the absence of *Y. enterocolitica* O:9 specific phages among 95 *Y. enterocolitica* phages isolated from 793 pig stool samples taken at 14 Finnish farms [29]. These observations support the need to isolate and characterize novel *Y. enterocolitica* bacteriophages in order to expand the repertoire of candidate phages for use in food safety applications. So far, however, few potential sources for phages have been considered, such as raw sewage, farm manure (pig manure), and soil. Moreover, given that the sampling of environmental phages seems to be influenced by the methodology employed [137]; there exists a large diversity of potentially useful phages left unexplored limited by the present isolation and propagation approaches.

### 5.2. Synthesis of RBPs

It is important also to highlight that the assembly pathways of RBPs may complicate their synthesis as functional ligands for use in diagnostics. For some RBPs, phage-encoded chaperone proteins participate in the assembly and disaggregation of supramolecular structures and are required for the correct folding and assembly of RBPs into homotrimers, which is essential for functional activity [138,139,140,141,142,143]. Notably, the mechanisms for protein folding were different for each of the three RBPs produced. RBPs Gp17 and Gp47 folded endogenously when expressed in *E. coli*, as has been reported for other Podoviruses [108,144]. However, the RBP protein sequence of phage vB_YenP_AP5 was found to contain an intramolecular chaperone (ICD) which mediates quaternary folding and auto-proteolysis, excising itself from the stable, mature trimer [103,145,146]. This mechanism was first described in phage proteins belonging to different but structurally similar protein families: bacteriophage endosialidases, capsule depolymerases, and appendage proteins [103,147,148,149,150]. This ICD domain is also present in other *Y. enterocolitica* infecting phages (φYeO3-12, vB_YenP_AP10, phiYe-F10, φR2-01), and thus, the synthesis of their respective RBP proteins is also readily attainable. In contrast, the long tail fiber protein Gp37 derived from the myovirus vB_YenM_TG1 was produced via simultaneous co-expression with the bacteriophage encoded chaperones Gp38 and Gp57A in a two-vector system as described for the expression of the long-tail fiber protein of phage T4 [151].

Several other protein-engineering strategies have also been proposed to circumvent the need for co-expression with chaperone proteins to guide the production of existing and as of yet unidentified potential RBP ligands that may be considered for use in detection assays. These approaches involve the use of foldons, carrier partners that promote proper folding, increase solubility, and enhance expression [152,153]. Among these, the T4 fibritin trimerization domain (aa 1–31) [154,155,156], the phage T4 trimerization motif of the structural protein Gp5 (aa 435–575) [157,158,159,160], and *E. coli* SLyD (aa 1-165) which performs chaperone functions [161,162], merit consideration.

### 5.3. Acceptance of Phages for Biocontrol

Although several phage-based products have been commercially available in the food industry since the regulatory acceptance of the first phage-based product in 2006, regulatory issues related to the application of phages in foods are still considered a major obstacle [4]. Regulatory authorities usually follow the existing regulations approved for ordinary drugs as there have been no guidelines established for regulatory approval of phages. Until now, there is no scientific evidence that phages themselves could impose a safety problem. In spite of this, regulatory authorities still consider phages as a potential risk. However, it is not necessary to maintain a negative perception of phages just because phages do not meet existing guidelines designed for drugs such as antibiotics. Therefore, a regulatory framework that is suitable for phages should be developed as soon as possible [4,163]. There is no doubt that further extensive research is required for the widespread use of phages. It is essential to establish adequate phage preparation methodologies as phage characterization, and purification are closely related to safety [164,165]. We are looking forward to the beginning of a new era of phages.

## Figures and Tables

**Table 1:** Bacteriophages infecting *Yersinia enterocolitica.*

Bacteriophage ^a^	Host Range	Host Receptor ^b^	Example Phage	References
*Podoviruses*				
φYeO3-12 [NC_001271.1]	*Y. enterocolitica* O:1; O:2; O:3	O-PS	Similar to *Enterobacteria* phage T7	[30]
vB_YenP_AP5 [KM253764.1]	*Y. enterocolitica* O:1; O:2; O:3	O-PS	Similar to *Enterobacteria* phage T7	[31,32]
phiYe-F10 [KT008108.1]	*Y. enterocolitica* O:3	O-PS	Similar to *Enterobacteria* phage T7	[33]
vB_YenP_AP10 [KT852574]	*Y. enterocolitica* O:1, O:2, O:3, O:6, O:6,31, O:35,36, O:41(27), K1 *Y. mollareti* O:3 *Y. frederiksenii* O:3 and O:35 *Y. intermedia* O:52,54	O-PS in O:3 strains	Similar to *Enterobacteria* phage T7	[31]
φR8-01 [HE956707.1]	*Y. enterocolitica* O:3 (O-PS and OC negative strains ^b^)	IC	Similar to *Enterobacteria* phage T7	[34]
φ80-18 [HE956710.1]	*Y. enterocolitica* O:8, O:7,8	O-PS	Similar to *Enterobacteria* phage T7	[35]
fPS Group 1 phages (11 closely related phages with phage fPS-7 [LT961840] as type species)	*Y. enterocolitica* O:3	O-PS	Similar to *Enterobacteria* phage T7	[28]
fPS Group 2 phages (fPS-53, fPS-85, fPS-89 fPS-54-ocr with phage fPS-53 [LT962379] as type species)	*Y. enterocolitica* O:3	OC could be the receptor for fPS-53, fPS-85, and fPS-89; probable Omp as a receptor for fPS-54-ocr	Similar to *Enterobacteria* phage T7	[28]
fPS Group 3 phages (phage fPS-59 [LT961845])	*Y. enterocolitica* O:3	O-PS	Similar to *Enterobacteria* phage T7	[28]
fHe-Yen3-01 [KY318515]	Broad host range infecting (29.2%, 31/106) of the *Yersinia* strains tested	Unknown	Similar to *Enterobacteria* phage T7	[36]
*Siphoviruses*				
φR2-01 [HE956708.1]	*Y. enterocolitica* O:8 (O-PS negative strains ^b^).	BtuB	Similar to *Enterobacteria* phageT5	[37]
PY-54 [NC_005069.1]	*Y. enterocolitica* O:5, O:5,27, Some *Y. enterocolitica* Biotype 1A strains	Unknown	Similar to phage λ	[38,39,40]
*Myoviruses*				
PY-100 [AM076770.1]	*Y. enterocolitica* O:3, O:5,27, O:8, O:9, some Biotype 1A strains, and some untypeable strains *Y. pestis Y. pseudotuberculosis Y. intermedia Y. kristensenii Y. frederiksenii Y. rohdei Y. mollareti*	Unknown	Similar to phiPLPE-like phages (Dwarf Myoviruses)	[41,42,43]
φR1-37 [NC_016163.1]	*Y. enterocolitica* O:1, O:3, O:5, O:5,27, O:6, O:6,31, O:9, O:21, O25,26,44, O41,43, O41(27)43, O:50. *Y. intermedia* O:52,54 *Y. similis*	OC in O:3	Similar to *Pseudomonas aeruginosa* phage φKZ	[41,42]
φR1-RT [HE956709.1]	*Y. enterocolitica* O:1, O:2, O:3, O:5, O:5,27, O:6, O:7,8, O:9	OmpF and IC of LPS	Similar to *Enterobacteria* phage T4	[44]
vB_YenM_TG1 [KP202158.1]	*Y. enterocolitica* O:1, O:2, O:3, O:5, O:5,27, O:6, O:7,8, O:9	OmpF and IC of LPS	Similar to *Enterobacteria* phage T4	[44]
fHe-Yen9-01 [KY593455]	Broad host range infecting 61.3%(65/106) of the *Yersinia* strains tested 65.4% (53/81) of the *Y. enterocolitica* strains tested	Unknown	Similar to *Enterobacteria* phage T4	[36]

^a^ GenBank accession numbers are listed in brackets; ^b^ LPS, a component found on the outer membrane of Gram-negative bacteria, is in *Y. enterocolitica* O:3 composed of lipid A (LA), inner core (IC), outer core (OC), and O-specific Polysaccharide (O-PS) [42].
